# Resection of Lung Cancer After COVID-19 in a Patient With Severe Chronic Obstructive Pulmonary Disease

**DOI:** 10.1016/j.atssr.2025.02.004

**Published:** 2025-03-03

**Authors:** Shumpei Kato, Takashi Sakai, Megumi Kusano, Satoshi Koezuka, Yoko Azuma, Akira Iyoda

**Affiliations:** 1 Division of Chest Surgery, Department of Surgery, Toho University School of Medicine, Tokyo, Japan

## Abstract

Evidence regarding the safety of thoracic surgery after COVID-19 is insufficient. The postoperative complication rate is high in patients with comorbidities, including chronic obstructive pulmonary disease, who undergo thoracic surgery. Herein we report a woman with advanced lung cancer associated with severe pulmonary dysfunction with a percentage of predicted forced expiratory volume in 1 second of 44.5% and percentage of predicted diffusion capacity of the lung for carbon monoxide of 38.9% due to chronic obstructive pulmonary disease detected after COVID-19. Curative resection was safely performed with perioperative management including respiratory physiotherapy, inhalation therapy, and adequate preoperative waiting period.

Surgeries immediately after resolution of COVID-19 are deemed risky. The guidelines recommend that elective surgery be performed because of high rates of mortality and respiratory complications in procedures performed earlier.^1^^,^^2^ Current decreases in the severity and thromboembolic risk of COVID-19 call for a reassessment of the waiting period,^3^ although the risk of perioperative complications is high, particularly in thoracic surgeries.^1^^,^^4^ In addition, although a smoking history is known to increase the risk of COVID-19 severity and mortality,^5^ the rates of mortality and respiratory complications are also high in patients with chronic obstructive pulmonary disease (COPD).^1^ Thus, the waiting periods for elective surgeries should not be absolute, but carefully considered for each case, taking into account preoperative risk assessment, surgical invasiveness, and comorbidities,^3^ and also being aware of cancer progression.

Herein, we report a patient with very poor (Global Initiative for Chronic Obstructive Lung Disease stage 3) lung function due to COPD and as a sequela of COVID-19, who underwent a successful lobectomy after her respiratory function improved with physiotherapy, inhalation therapy, and an appropriate waiting period and treatment for COVID-19.

A 65-year-old woman was referred to our hospital for the treatment of a lung tumor (Figure 1A). She had been treated for multiple collagen vascular diseases, including systemic lupus erythematosus and Behçet disease, and was a current smoker with a 44 pack-year history. Chest computed tomography showed a 10-cm tumor in the right lower lobe (Figure 1B). Because she had COVID-19 of moderate severity at the first visit, she was treated with oral antiviral medication without hospitalization. Three weeks after confirmation of her infection, a transbronchial tumor biopsy was performed and she was diagnosed with squamous cell carcinoma, cT4N1M0 stage IIIB. The treatment plan was initial surgery as she could not receive immunotherapy due to her collagen vascular diseases and the image findings confirmed that the tumor was resectable.

**Figure 1:**
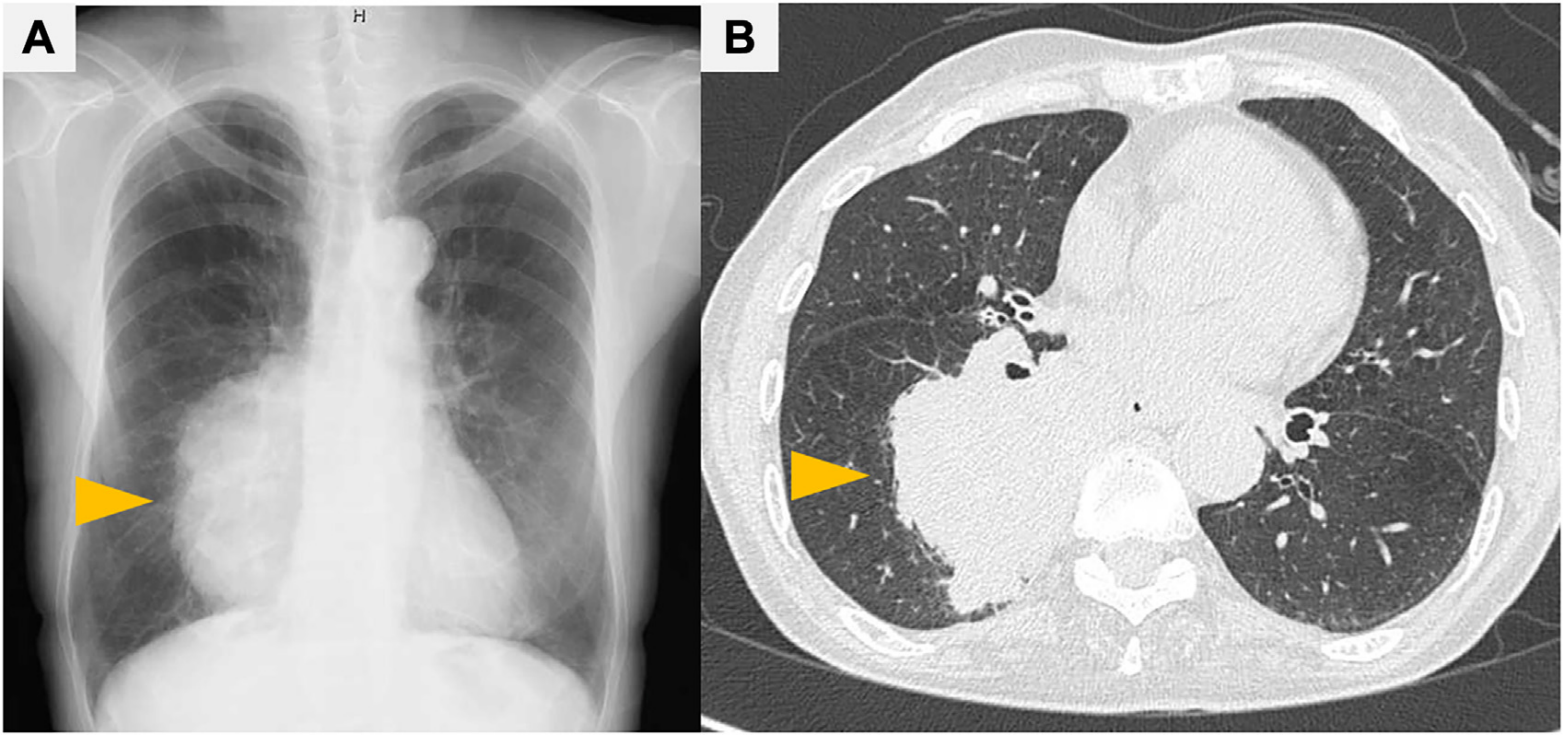
Perioperative imaging. (A) Chest radiography revealing a huge lung tumor in the right lung field (arrowhead). (B) Computed tomography showing a >10-cm tumor in the right lower lobe (arrowhead).

Pulmonary function tests revealed a forced expiratory volume in one second (FEV_1_) of 850 mL, a FEV_1_/forced vital capacity (FVC) of 46.7%, a percentage of predicted FEV_1_ (FEV_1_%pred) of 44.5%, and a percentage of predicted diffusion capacity of the lung for carbon monoxide (D_lco_%pred) of 38.9%. The results indicated that the patient had both obstructive and diffusion impairments caused by severe COPD and the sequelae of COVID-19. The predicted postoperative FEV_1_ (ppo%FEV_1_) and predicted postoperative D_lco_ (ppo%D_lco_) after right lower lobectomy were 32.8% and 28.7%, respectively, which was considered a high risk for postoperative respiratory failure.

Thus, the patient’s COPD was treated by smoking cessation, respiratory physiotherapy, and inhalation therapy consisting of a long-acting muscarinic antagonist/long-acting beta-agonist. Similarly, respiratory rehabilitation was performed for post-COVID-19 sequelae. Four weeks later, her respiratory function had improved, with an FEV_1_ of 1370 mL, an FEV_1_%pred of 72.4%, and a D_lco_%pred of 44.6%. Her ppo%FEV_1_ was 53.3% and ppo%D_lco_ was 32.8%. The findings suggested that the right lower lung lobectomy could be performed safely, and the patient underwent surgery 6 weeks post-infection (Figure 2).

**Figure 2:**
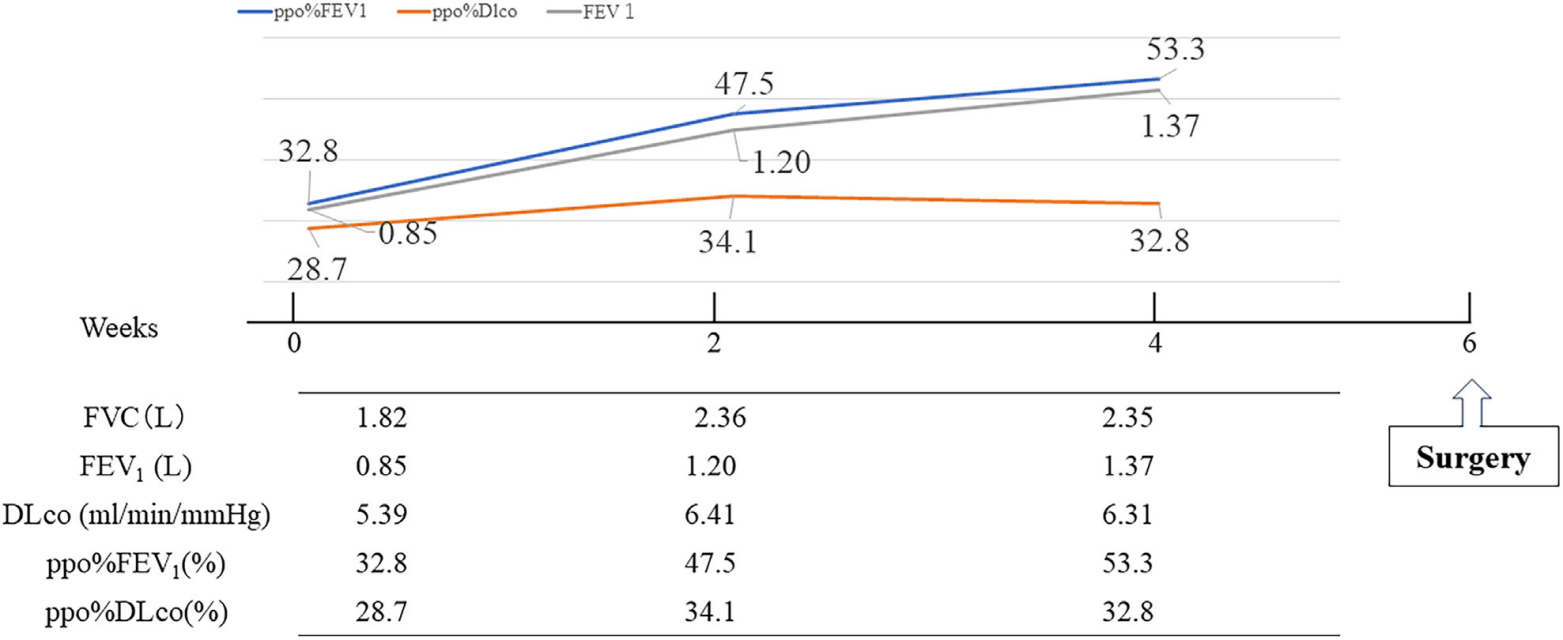
Chart of pulmonary function. Initial pulmonary function tests revealed both obstructive and diffusion impairments due to severe chronic obstructive pulmonary disease and as sequelae of COVID-19. Smoking cessation, respiratory physiotherapy, and inhalation therapy were effective, leading to improved respiratory function at 2 and 4 weeks of treatment. (DLco, diffusing capacity of lung for carbon monoxide; FEV_1_, forced expiratory volume in 1 second; FVC, forced vital capacity; ppo%FEV_1_, predicted postoperative FEV_1_; ppo%DLco, predicted postoperative DLco.)

Although the tumor had invaded the chest wall, which necessitated additional parietal pleura resection, complete resection was achieved via thoracotomy. The histopathologic diagnosis was a squamous cell carcinoma exceeding 7 cm, with partial invasion into the parietal pleura and a single mediastinal lymph node metastasis (no. 7) out of 22 resected, pT4N2M0 stage IIIB. The patient was discharged on postoperative day 6 without any complications. Pulmonary function tests were reassessed 3 months postoperatively: Her FEV_1_%pred was 62.6% and a D_lco_%pred was 34.7%, showing better function compared with the preoperative predicted values. The patient underwent 4 cycles of adjuvant chemotherapy with cisplatin and vinorelbine and was doing well at 12 months after surgery.

## Comment

Elective surgery is recommended for patients after resolution of COVID-19 to prevent the spread of infection to the medical staff and postoperative complications. Based on a worldwide cohort study showing a high mortality rate and respiratory complications after surgery for patients with COVID-19, guidelines have recommended that elective surgeries be performed a minimum of 7 weeks after resolution of the infection, and for asymptomatic or mild infections, at least 4 weeks.^2^^,^^6^ However, the current guidelines have recommended reducing the waiting periods because the severity and mortality rates of COVID-19 have decreased.^3^

The guidelines also have recommended that waiting periods should be based on individual patients according to their residual symptoms and comorbidities, along with consideration of the risk for delaying surgery for a condition that requires it. Comorbidities such as diabetes and cardiovascular disease have been reported to be associated with the severity of COVID-19 infection and perioperative mortality. Coexisting respiratory disease is a major risk of severe disease.^5^ COPD has been identified as a contributing factor to the severity of COVID-19, and in patients with lung cancer who have the disease, a smoking history has been reported to affect mortality rates.^5^

Patients requiring thoracic surgery often have a smoking history and underlying lung diseases like emphysema, making preoperative treatment important for reducing the risk of complications.^7^ Because we were able to perform thoracic surgery safely after administering long-acting muscarinic antagonist/long-acting beta-agonist inhalation therapy to our patient,^8^ we believe that preoperative physiotherapy and inhalation medication should be considered for a patient with emphysema who needs surgery.

The indiscriminate establishment of waiting periods can pose a risk for progression in lung cancer. Our patient had a large lung tumor with a metastatic lymph node, which necessitated prompt intervention. The patient's squamous cell carcinoma was mutation-negative and had low expression of programmed death-ligand 1, and she had concomitant collagen vascular disease, making preoperative therapy challenging. Thus, she needed to undergo surgery. Fortunately, physiotherapy and inhalation therapy were effective, allowing for early surgical treatment. Prompt treatment intervention in cases of underlying lung disease and poor lung function post-COVID-19 infection can enable feasible cancer treatments.
